# Reply to: “Is Prolonged Intermittent Renal Replacement Therapy actually safe for hemodynamically unstable patients?”

**DOI:** 10.1186/s13054-023-04459-w

**Published:** 2023-04-29

**Authors:** Edward G. Clark, Anitha Vijayan

**Affiliations:** 1 Division of Nephrology, Department of Medicine, University of Ottawa, Ottawa, Canada; 2 Division of Nephrology, Washington University in St. Louis, St. Louis, MO USA

Dear Editor,

We thank Dr. Honoré and his colleagues for their comments on our brief review entitled “How I Prescribe Prolonged Intermittent Renal Replacement Therapy” [1]. Their concern that accumulation of the small amount of acetate used as the pH-stabilizing factor in standard bicarbonate dialysate (3–7 mM) limits the hemodynamic tolerability of some forms of prolonged intermittent renal replacement therapy, such as sustained low-efficiency dialysis (SLED), merits further investigation. However, the evidence that acetate accumulation is a clinically important issue is limited. For example, it should be noted that the article cited to indicate that up to 11% of SLED treatments are discontinued due to hypotension only reported that one of 234 SLED sessions (0.4%) were discontinued for that reason. This reportedly occurred during one of 110 SLED sessions [0.9%] in 35 patients randomized to acetate-containing dialysate) [2]. The extent to which this relatively small single-center trial with low methodologic quality can inform clinical practice is minimal. While Dr. Honoré and colleagues additionally highlight theoretical evidence suggesting how low concentrations of acetate in dialysate might precipitate hemodynamic instability, other experts have suggested that any hemodynamic benefit observed in studies assessing the use of acetate-free biofiltration “may be due to additional thermal cooling, or a more gradual change in potassium and other electrolytes, rather than simply due to removal of acetate from the dialysis fluids” [3].

While we acknowledge that renal replacement therapy (RRT) modality comparison studies are fraught with methodologic challenges, a 2021 systematic review of studies involving hemodynamically unstable patients with acute kidney injury (*N* = 1160, in total) concluded that there was no major advantage to using continuous renal replacement therapy (CRRT) versus SLED [4]. Beyond that, a recent systematic review and network meta-analysis of randomized controlled trials comparing RRT modalities in critically ill patients with acute kidney injury (including five trials comparing CRRT vs SLED [*N* = 463]) determined that SLED may be the most effective intervention at reducing mortality (albeit with a low certainty of evidence) and, more robustly, that it is non-inferior to CRRT [5].

The potential issue of low-dose acetate accumulation in patients receiving SLED could ultimately prove to be clinically relevant. The study suggesting this [2] should be attempted to be replicated. Nonetheless, based on the current evidence and our clinical experience, we assert that prolonged intermittent renal replacement therapy, including SLED, should be considered a safe option for hemodynamically unstable patients.

## Data Availability

Not applicable.
